# Intratumoral Lentivector-Mediated TGF-β1 Gene Downregulation As a Potent Strategy for Enhancing the Antitumor Effect of Therapy Composed of Cyclophosphamide and Dendritic Cells

**DOI:** 10.3389/fimmu.2017.00713

**Published:** 2017-06-30

**Authors:** Joanna Rossowska, Natalia Anger, Agnieszka Szczygieł, Jagoda Mierzejewska, Elżbieta Pajtasz-Piasecka

**Affiliations:** ^1^Ludwik Hirszfeld Institute of Immunology and Experimental Therapy, Polish Academy of Sciences, Wroclaw, Poland

**Keywords:** lentivector, TGF-β1 silencing, dendritic cells, MC38 colon carcinoma, cyclophosphamide, tumor environment reprogramming, antitumor immunotherapy

## Abstract

Vaccination with dendritic cells (DCs) stimulated with tumor antigens can induce specific cellular immune response that recognizes a high spectrum of tumor antigens. However, the ability of cancer cells to produce immunosuppressive factors drastically decreases the antitumor activity of DCs. The main purpose of the study was to improve the effectiveness of DC-based immunotherapy or chemoimmunotherapy composed of cyclophosphamide (CY) and DCs by application of lentivectors (LVs)-encoding short hairpin RNA specific for TGF-β1 (shTGFβ1 LVs). We observed that s.c. inoculation of both MC38 cells with silenced expression of TGF-β1 (MC38/shTGF-β1) and direct intratumoral application of shTGFβ1 LVs contributed to reduction of suppressor activity of myeloid cells and Tregs in tumor. Contrary to expectations, in mice bearing wild tumor, the application of shTGFβ1 LVs prior to vaccination with bone marrow-derived DC stimulated with tumor antigens (BMDC/TAg) did not influence myeloid-derived suppressor cell (MDSC) infiltration into tumor. As a result, we observed only minor MC38 tumor growth inhibition (TGI) accompanied by systemic antitumor response activation comparable to that obtained for negative control (shN). However, when the proposed scheme was complemented by pretreatment with a low dose of CY, we noticed high MC38 TGI together with decreased number of MDSCs in tumor and induction of Th1-type response. Moreover, in both schemes of treatment, LVs (shTGFβ1 as well as shN) induced high influx of CTLs into tumor associated probably with the viral antigen introduction into tumor microenvironment. Concluding, the application of shTGFβ1 LVs alone or in combination with DC-based vaccines is not sufficient for long-lasting elimination of suppression in tumor. However, simultaneous reduction of TGF-β1 in tumor microenvironment and its remodeling by pretreatment with a low dose of CY facilitates the settlement of peritumorally inoculated DCs and supports them in restoration and activation of a potent antitumor response.

## Introduction

Cancer immunotherapy using dendritic cells (DCs) has been one of the leading research topic since efficient tumor antigen presentation was determined to be a key process in inducing a specific antitumor response (1, 2). Vaccination with dendritic cells stimulated or transfected with whole tumor antigens can induce specific cellular immune response that recognizes a high spectrum of tumor antigens; however, the ability of cancer cells to produce immunosuppressive factors drastically decreases the antitumor effectiveness of DC-based vaccines (3–5). A lot of attention was paid to the TGF-β anti-inflammatory cytokine, since its increased secretion in the tumor microenvironment is often correlated with poor prognosis in patients (6). This dualistic nature of TGF-β is well described in the literature (7). During early stages of carcinogenesis, this cytokine suppresses tumor cell proliferation, angiogenesis, and inflammation, as well as induces apoptosis and autophagy (8, 9). On the other hand, in cancer progression, TGF-β gains tumor-promoting functions. Tumor cells are capable of using TGF-β in order to promote growth factor production, differentiation toward a malignant phenotype, and induce epithelial-mesenchymal transition, which leads to increased invasiveness of the tumor and creation of metastases (10). Apart from the aforementioned processes, the cytokine is also responsible for a broad array of other tumorigenic effects, i.e., dysregulation of cyclin-dependent kinase inhibitors, introduction of changes in the architecture of the cytoskeleton, as well as aberrations in the formation of the extracellular matrix (9). One of the most pivotal functions of TGF-β is its ability to modulate the components of immune response. The cytokine plays a key role in hindering of activation, maturation, and differentiation of both innate and adaptive immune cells. Furthermore, it influences the overall immune tolerance by regulating the differentiation and induction of regulatory T cells (6). TGF-β may also interfere with functioning of DCs by impairing their ability to migrate to secondary lymphoid organs and successfully activate naïve T lymphocytes, as well as by downregulation of cell surface MHC molecules, costimulatory molecules (CD80, CD40, and CD86 in mice), and chemokine receptors (11, 12). Due to its strong immunosuppressive effect on immune cells and high protumorigenic potential, TGF-β is regarded as a promising target in antitumor therapy. Many effective anti-TGF-β drugs are currently being developed, some of which are already being tested in pre-clinical studies and clinical trials. Most notably, the usage of TGF-β neutralizing antibodies, TGF-β receptor kinase inhibitors, as well as TGF-β anti-sense oligonucleotides and siRNAs in potential treatments was described. Finally, it was shown that TGF-β signaling inhibitors are generally safe and can be effectively adapted for clinical applications (13–17).

Lentivectors (LVs) constitute another interesting perspective for inhibition of TGF-β expression in cells. They are a highly efficient gene delivery tool capable of inducing stable and long-term gene silencing, by expression of short hairpin RNAs (shRNAs). Experiments have shown that LVs may be safer and less mutagenic than retroviral vectors, because they do not insert close to cell cycle genes or oncogenes (18, 19). The third-generation lentiviral systems contain self-inactivating promoters and are devoid of any viral proteins responsible for pathogenicity of the viruses. This mechanism increases the safety of application of LVs and decreases their immunogenicity (20).

After having taken into consideration the suppressive effect of TGF-β on dendritic cells, as well as the lowered immunogenicity of the new generation of LVs, in our study, we have decided to use LVs carrying shRNA sequences specific for TGF-β1 as components of a DC-based immunotherapy and chemoimmunotherapy, which consisted of low-dosage cyclophosphamide (CY) and DC-based vaccines. Although, in mammalian cells three distinct isoforms of TGF-β (TGF-β1-3) are expressed, we have selected the TGF-β1 isoform due to its prominently elevated expression in highly aggressive and metastatic tumors, as well as its strong immunosuppressive effects (13, 21, 22). The main purpose of the study was to improve the effectiveness of DC-based immunotherapy and chemoimmunotherapy through attenuation of TGF-β1-induced immunosuppression. The LVs were used to obtain genetically modified MC38 cell lines with silenced expression of TGF-β1 and to reduce the production of TGF-β1 *in situ* by intratumoral inoculation. The gathered data indicate that MC38 cells with silenced expression of TGF-β1 were characterized by increased immunogenicity *in vitro*. On the other hand, the s.c. inoculation of both MC38 cells with silenced expression of TGF-β1 (MC38/shTGF-β1) and direct intratumoral application of shTGFβ1 LVs contributed to reduction of suppressor activity of myeloid cells and Tregs in tumor. Additionally, intratumoral injections of LVs encoding shRNA specific for TGF-β1 after pretreatment with CY significantly increased the effectiveness of DC-based therapies in mice bearing MC38 colon carcinoma. The tumor growth delay was accompanied by reduced number of myeloid-derived suppressor cell (MDSC) cells infiltrating tumor and enhanced systemic antitumor response.

## Materials and Methods

### Mice

Female C57BL/6 mice were obtained from the Center for Experimental Medicine of the Medical University of Bialystok (Bialystok, Poland). All experiments were performed according to the properly established protocols and were approved by the 1st Local Ethics Committee for Experiments with the Use of Laboratory Animals, Wroclaw, Poland.

### Cell Culture

MC38 murine colon carcinoma cell line (23) was maintained in RPMI 1640 (Gibco) supplemented with 100 U/ml penicillin (Polfa), 100 mg/ml streptomycin (Polfa), 0.5% sodium pyruvate (Sigma-Aldrich), 2-mercaptoethanol (Sigma-Aldrich) named here as complete medium (CM), and 5% of fetal bovine serum (FBS, Sigma-Aldrich). Lenti-X™ 293T cell line (Clontech) was maintained in high-glucose Dulbecco’s Modified Eagle Medium (Gibco) supplemented with 100 U/ml penicillin, 100 mg/ml streptomycin, 0,5% sodium pyruvate, and 10% of FBS. MC38 and LentiX cells were cultured in 75 cm^2^ flasks (Corning) and passed every 2–3 days. Dendritic cells [bone marrow-derived dendritic cells (BMDC)] were differentiated *ex vivo* from bone marrow of C57BL/6 mice according to the procedure described in our previous articles (24). BMDCs were cultured in CM supplemented with 10% of FBS (Sigma-Aldrich), recombinant murine GM-CSF (40 ng/ml, ImmunoTools), and recombinant murine IL-4 (10 ng/ml, ImmunoTools). On the 6th day, loosely attached immature dendritic cells were collected and used in further *in vitro* experiments or utilized as a BMDC-based vaccine.

### Lentiviral Vector Production

**Figure 1:**
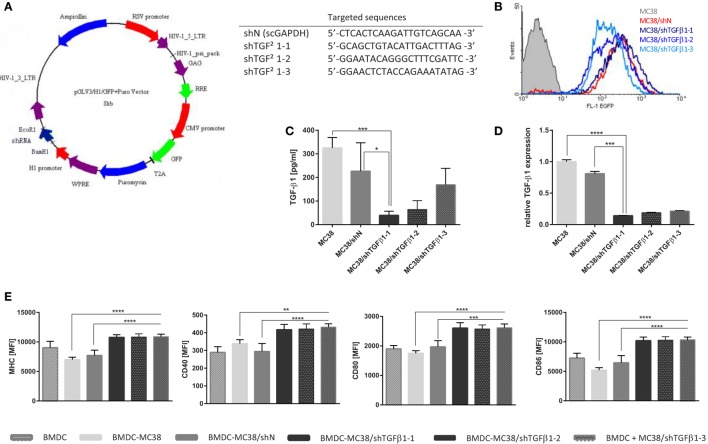
shTGF-β1 sequence activity in stable transduced MC38 cell line. **(A)** Scheme of lentiviral vector utilized to silence of TGF-β1 gene experession and sequences of tested short hairpin RNAs (shRNAs); **(B)** EGFP expression in MC38 cells transduced with lentiviral vectors carrying shRNA against TGF-β-1 or scrambled sequence against GAPDH as a negative control after 2 weeks selection with puromycin vs. wild MC38 cells; **(C)** TGF-β1 concentration in supernatant collected after 24 h culture of transduced MC38 cells measured using ELISA assay; **(D)** Expression of TGF-β1 mRNA in MC38 cells transduced with shTGF-β1 or shN vs. wild type of MC38; **(E)** The stage of bone marrow-derived dendritic cell (BMDC) differentiation after stimulation with MC38/shTGF-β1 cells. A mean fluorescence intensity (MFI) corresponds to expression level of particular molecule. The results are expressed as a mean ± SD calculated for three repeats in two or three independent experiments. The differences between groups were estimated using non-parametric Kruskal–Wallis test followed by Dunn’s multi comparison test (*p < 0.05, **p < 0.01, ***p < 0.001, ****p < 0.0001).

Lentiviral vectors were produced using the third-generation lentiviral system consisting of pMDLg/pRRE, pRSV-Rev, pMD2.G [the plasmids were a gift from Didier Trono (Addgene plasmid # 12251, 12253, 12259)] and expression plasmids pGLV-H1-GFP + Puro (EzBiolab). The expression plasmids encoded three different shRNA sequences against TGF-β1. The control vector encoded scrambled sequence of shRNA against human GAPDH (shN). Map of the expression plasmid and sequences of shRNA are presented in Figure 1. Lentiviral vectors were produced and concentrated according to the protocol established by Kutner RH and coworkers (25) with some modifications. Briefly, 80% confluent Lenti-X cells, cultured in 150 cm^2^ plates, were co-transfected with the pGLV-H1-GFP + Puro expression plasmid, a plasmid encoding a VSV virus coat protein (pMD2.G), the packaging vector (pMDL-g/p-RRE), and the vector of the REV protein gene (pRSV-REV). After 24 h, culture medium was replaced with Opti-MEM + GlutaMAX (Gibco) supplemented with penicillin, streptomycin, and 5% of FBS. After 48 h, lentiviral vector-containing supernatant was collected, centrifuged, and passed through 0.45 µm PES filters (Millipore). Next, supernatant was mixed with PEG 6000 (Sigma-Aldrich), 4 M NaCl, and PBS, incubated, and centrifuged using Beckman Coulter JA-10 rotor at 5,000 rpm. Pellet of lentiviral vectors was suspended in small volume of PBS and stored at −80°C. The titer of the lentiviral vectors was determined by serial dilution method using MC38 cells. The LVs were used for establishment of new MC38 cell lines with silenced expression of TGF-β1 as well as were applied as one of the components of antitumor therapy.

### Characteristics of MC38 Cell Lines with Silenced Expression of TGF-β1

MC38/shTGFβ1-1, 2, 3, and MC38/shN cells were selected by culturing in CM supplemented with puromycin (10 µg/ml, Gibco). The intensity of EGFP expression was evaluated using FACS Calibur (Becton Dickinson). Total RNA from cultured MC38 cells was isolated using Nucleospin RNA isolation kit (Mecherey-Nagel) and reverse-transcribed with RevertAid First Strand cDNA Synthesis Kit (ThermoFisher) according to manufacturer instruction. Real-Time PCR was performed in triplicates using TaqMan^®^ Universal PCR Master Mix and TaqMan^®^ Gene Expression Assay primers for TGF-β1 (Applied Biosystem) according to manufacturer protocol. As a reference gene, mHPRT was used. Expression level of TGF-β1 in genetically modified cells was evaluated relatively to MC38/0 cells. To evaluate the tumorigenic activity of genetically modified cell lines, 8- to 10-week-old female C57BL/6 mice were subcutaneously inoculated in right flank with MC38/0, MC38/shN, MC38/shTGFβ1-1, MC38/shTGFβ1-2, or MC38/shTGFβ1-3 cells (1.1 × 10^6^/0.2 ml/mouse). Tumor volume was monitored every 2–3 day of the experiment. On the 30th day of the experiment, mice were sacrificed and their spleens and tumors were dissected, homogenized, and frozen in liquid nitrogen for further analyses.

### Stimulation of Immature Dendritic Cells with MC38 Cells with Silenced Expression of TGF-β1

Immature BMDC were cocultured with mitomycin C-treated MC38/0, MC38/shN, and MC38/shTGFβ1-1, 2, 3 cells in the presence of GM-CSF (40 ng/ml). After 24 h, dendritic cells were harvested and labeled with monoclonal antibodies conjugated with fluorochromes (BD Biosciences): anti-CD40 PE, anti-CD80 APC, anti-CD86 PE-Cy7, anti-MHC II FITC, anti-CD11b PerCP-Cy5.5, and anti-CD11c BV650. The expression of cell surface markers was analyzed using FACS Calibur with CellQuest software (Becton Dickinson).

### Intratumoral Activity of LVs

8- to 10-week-old female C57BL/6 mice were subcutaneously inoculated in right flank with MC38/0 cells (1.1 × 10^6^/0.2 ml/mouse). On the 14th, 15th, and 17th day of the experiment, mice were injected i.t. with lentiviral vectors encoding shRNA against TGF-β1 (shTGFβ1-1, 2 × 10^6^ TU/50μl/mouse). As a control, mice received lentiviral vectors encoding scrambled shRNA against human GAPDH (shN). Two days after the third injection, mice were sacrificed and their tumor nodules were dissected for analysis of lentiviral transduction efficacy. Tumors were homogenized and divided for tumor cell culture and flow cytometry analysis. Production of TGF-β1 by cells isolated from tumors was estimated by ELISA in supernatant collected from 24 h culture of 5 mg tumor tissue/ml. The percentage of transduced cells expressing EGFP was measured during flow cytometry analysis of cells isolated from tumors. Additionally, cells were stained with cocktail of fluorochrome-conjugated antibodies for myeloid cells identification: CD45 V500, CD3 PE-CF594, CD19 PE-CF594, CD49b PE-CF594 (all from BD Biosciences), CD11b PerCP-Cy5.5, CD11c BV650, F4/80 AlexaFluor 700, Ly6C BV510, Ly6G BV605, and MHC II APC-Cy7, CD86 PE-Cy7 (all from Biolegend) and cocktail for lymphocytes identification: CD45 BV605, CD3 BV650, CD4 FITC, CD8 APC-Fire, CD25 PE, CD44 PE-Cy7, and CD62L PerCP-Cy5.5 (all from Biolegend). Next, cells stained with lymphocyte cocktail were fixed with Foxp3/Transcription Factor Staining Buffer Set (eBioscience) and incubated with anti-FoxP3 APC (eBioscience). The analysis of myeloid and lymphocyte populations in tumors was carried out using LSR Fortessa with Diva software (Becton Dickinson).

### Therapeutic Treatment Schedule

8- to 10-week-old female C57BL/6 mice were subcutaneously inoculated in right flank with MC38/0 cells (1.1 × 10^6^/0.2 ml/mouse). On the 14th day of the experiment, mice were injected i.t. with lentiviral vectors encoding shRNA against TGF-β1 (shTGFβ1-1, 2 × 10^6^ TU/50 μl/mouse). As a control, mice received lentiviral vectors encoding scrambled shRNA against human GAPDH (shN) or concentrated Lenti-X culture medium (PEG). On the 15th day, tumor antigen-pulsed dendritic cell-based vaccines were applied p.t. (BMDC/TAg, 2 × 10^6^/0.2 ml/mouse or 0.5 × 10^6^/0.2 ml/mouse). LVs and BMDC-based vaccines were applied three times with 1-week intervals. BMDC were stimulated with MC38 tumor lysate prepared according to procedure described in our previous article (26). On the 36th day, 1 week after the third DC-based vaccination, six mice from each group were sacrificed, and their spleens and tumor nodules were dissected, homogenized, and stored in liquid nitrogen for further analyses. Depending on the experiment, mice were additionally injected i.p. with CY (Baxter, 150 mg/kg body weight) on the 12th day. During the experiments, mice with growing tumors were monitored every 2–3 days, and the tumor volume was estimated according to the formula *a/*2 *× b*^2^, where *a* represents the largest and *b* the smallest tumor diameter. The therapeutic effect of the treatment was evaluated using tumor growth inhibition (TGI), the percentage of tumor growth delay compared to the tumors of untreated mice Statistical differences were calculated using Friedman and Dunn’s multicomparison tests.

### Analysis of Lymphocyte Populations in Tumors of Mice after the Therapy

Tumor cells obtained from control or treated mice were thawed, centrifuged, and stained in one-step test using monoclonal antibodies conjugated with fluorophores: anti-CD45 PE-Cy7, anti-CD4 APC, anti-CD8 FITC, and anti-CD49b PE (all from BD Biosciences). In order to eliminate dead cells during the analysis, cells were additionally stained with DAPI dye (Molecular Probes). The analysis was carried out using LSR Fortesssa with Diva software (Becton Dickinson). In order to determine the percentage of T regulatory lymphocytes in tumors, the cells were stained with monoclonal antibodies: anti-CD45 PE-Cy7 (BD Biosciences), anti-CD4 FITC (eBioscience), and anti-CD25 PE (eBioscience). Then, cells were fixed using Foxp3/Transcription Factor Staining Buffer Set (eBioscience) according to manufacturer instruction and incubated with anti-FoxP3 monoclonal antibody conjugated with APC (eBioscience). The analysis was carried out using LSR Fortessa with Diva software (Becton Dickinson).

### Analysis of MDSCs in Spleen and Tumors of Mice after the Therapy

Spleen and tumor cells obtained from control or treated tumor-bearing mice were thawed, centrifuged, and incubated with monoclonal antibodies conjugated with fluorophores: anti-CD45 V500, anti-CD11b PerCP-Cy5.5, anti-CD11c BV605, anti-CD4 APC, anti-B220 APC, anti-CD49b APC, anti-Ly6C PE, anti-Ly6G APC-Cy7, anti-MHCII FITC, and anti-CD86 PE-Cy7 (all from BD Biosciences). After incubation, cells were suspended in PBS with DAPI dye (Molecular Probes) and analyzed using LSR Fortessa with Diva Software (Becton Dickinson) according to the procedure described by Rossowska and coworkers (27).

### Determination of Suppressor Activity of Spleen and Tumor-Derived MDSCs

Suppressor activity of spleen and tumor-derived MDSCs was evaluated using CFSE proliferation assay. Spleen and tumor cells obtained from tumor-bearing mice were labeled with monoclonal anti-CD11b antibodies conjugated with magnetic nanoparticles (BD Imag). CD11b^+^ cells were magnetically sorted and cocultured in a 1:1 ratio with splenocytes from healthy mice labeled with CFSE dye (15 min, 37°C; Molecular Probes) for 3 days in the presence of concanavalin A (2 µg/ml; Sigma-Aldrich) and murine IL-2 (100 LU/ml). After that, cells were harvested and stained with anti-CD4 APC and anti-CD8 PE-Cy7 monoclonal antibodies (BD Biosciences). The intensity of CFSE fluorescence in CD4^+^ and CD8^+^ cells was measured with FACS Calibur apparatus with CellQuest software.

### Analysis of Antitumor Response of Effector Spleen Cells

Spleen cells obtained from control or treated tumor-bearing mice were cocultured with mitomycin C-treated MC38 cells (50 mg MitC/3 × 10^6^ cells; 30 min., 37°C) in the presence of recombinant human IL-2 (200 U/ml). After 5 days of restimulation, supernatants were collected and stored in 4°C. Cytotoxic activity of cells with application of DiO lipophilic dye (Molecular Probes) were analyzed according to previously described procedure (28). The percentage of dead double positive (DiO^+^PI^+^) MC38 cells was determined after analysis using FACS Calibur with CellQuest software (Becton Dickinson). In order to determine the percentage of CD107^+^ cells, restimulated spleen cells were incubated for 2 h with MC38 cells in the presence of monoclonal anti-CD107 antibody conjugated with APC (BioLegend). After that, cells were stained with anti-CD8 PE-Cy7 and anti-CD49b PE and analyzed using LSR Fortessa with Diva software (Becton Dickinson).

### Determination of Cytokine Production

Production of cytokines by BMDC and restimulated spleen cells was evaluated using commercially available ELISA kits (IL-10, IL-4—BD Biosciences; IFN-γ, TGF-β1—eBioscience) according to manufacturer instruction.

### Statistics

All data were analyzed using GraphPad Prism 6 software. The statistical significance in kinetics of tumor growth was calculated using Friedman test followed by Dunn’s multi comparison *post hoc* test. In all remaining analyses, the statistical differences were calculated using the non-parametric Kruskal–Wallis test for multiple independent groups followed by Dunn’s multi comparison *post hoc* test. Differences with a *p*-value < 0.05 were regarded as significant.

## Results

### MC38 Murine Colon Carcinoma Cells with Silenced Expression of TGF-β1 Have Increased Potential to Induce Dendritic Cell Maturation and Their Proinflammatory Properties *In Vitro*

In the first step of the study, the effectiveness of three different shRNAs designed for silencing of murine TGF-β1 expression was evaluated. The map of the lentiviral vector encoding TGF-β1-targeting or control sequences of shRNAs were presented in the Figure 1A. New cell lines were characterized by high expression of EGFP (Figure 1B) and significantly reduced expression of TGF-β1. The Real-Time PCR (Figure 1D) as well as ELISA (Figure 1C) analyses confirmed that the sequence of shTGFβ1-1 was the most effective in silencing of TGF-β1. In order to check the immunogenicity of MC38 cell lines with silenced expression of TGF-β1, the influence of the cells on the activity of BMDCs was estimated. BMDCs stimulated with MC38/shTGFβ1 cells showed significantly increased expression of costimulatory molecules (CD40, CD80, CD86) as well as MHC class II compared to control cells incubated with MC38/shN or untransduced MC38 cells (Figure 1E). Although, no differences between individual transductants with silenced expression of TGF-β1 were observed, the obtained data showed that only the cells with reduced ability to produce TGF-β1 effectively differentiated immature dendritic cells into their mature stage, giving them the opportunity for potent presentation of tumor antigens. On the base of obtained results, we have chosen the shTGFβ1-1 for further analyses.

### MC38 Cells with Silenced Expression of TGF-β1 Are Unable to Induce Myeloid Cell-Derived Suppression *In Vivo*

**Figure 2:**
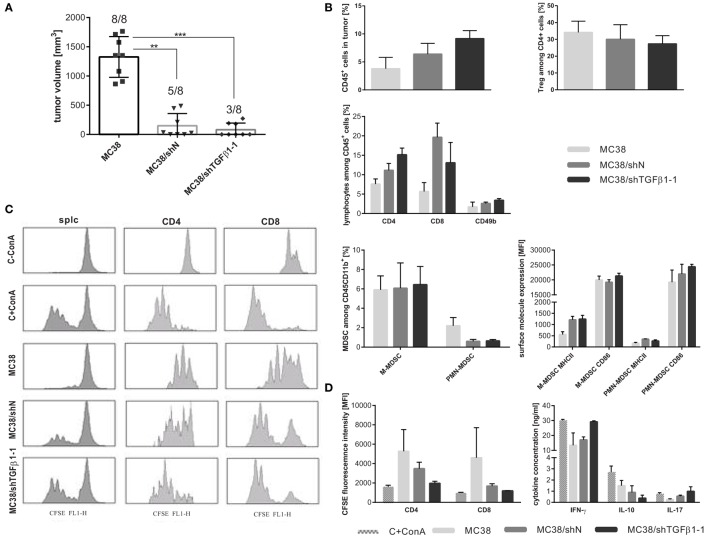
Immunogenicity of selected MC38 cells with silenced expression of TGF-β1 *in vivo*. **(A)** *MC38/shTGF*β*1-1, MC38*/shN, and unmodified MC38 cells (1.1 × 10^6^ cell/mouse) were injected s.c., and their ability to form a tumor nodule was estimated. Boxes present the mean tumor growth on the 32nd day after tumor cell injection. To calculate the mean ± SD at least eight mice per group were analyzed. The differences between groups were estimated using non-parametric Kruskal–Wallis test followed by Dunn’s multi comparison test (**p < 0.01, ***p < 0.001). **(B)** Characteristic of immune cells infiltrating into growing tumors collected on the 32nd day after injection of MC38/shTGFβ1-1 cell line and adequate negative controls. **(C,D)** Suppressor activity of spleen-derived myeloid-derived suppressor cell (MDSC) estimated using CFSE-based proliferation assay. Splenocytes from healthy mice stained with CFSE were cocultured with magnetically sorted CD11b^+^ cells from spleen of treated mice in the presence of ConA and IL-2 for 72 h. After coculture of CD11b^+^ cells with splenocytes, the T cell proliferation was measured by CFSE dilution. The mean fluorescence intensity (MFI) of whole population of splenocytes as well as proliferating CD8^+^ and CD4^+^ cells were presented as representative histograms **(C)** and bar graphs **(D)**. As a positive and negative control CFSE-labeled splc cultured with or without ConA were used. Concentrations of IFN-γ, IL-10, and IL-17 in supernatants collected after 72 h coculture of CD11b^+^ cells with splenocytes were measured using ELISA. **(B–D)** To calculate the mean ± SD, the analysis for three mice/group were performed.

To evaluate the effect of TGF-β1 gene silencing on the MC38 tumor growth *in vivo*, MC38/shTGFβ1-1 cells with the highest reduction of TGF-β1 expression were inoculated s.c. into the right flank of C57BL/6 mice. The rate of MC38/shTGFβ1-1 tumor growth was significantly slower when compared to untransduced MC38 cells. However, high reduction of tumorigenicity was observed also in the case of MC38/shN control cell line. On the 32nd day after tumor cell inoculation, the mean volume of MC38/shTGFβ1-1 tumors was 80 mm^3^ and five of eight mice rejected tumors, whereas the mean volume of MC38/shN tumors amounted to 150 mm^3^ and three of eight mice rejected tumors (Figure 2A). At the same time, untransduced MC38 cells formed tumor nodules with mean volume of 1,330 mm^3^.

Flow cytometric analysis of immune cells infiltrating tumors showed high influx of leukocytes (CD45^+^ cells) into MC38/shTGFβ1-1 tumors, which was accompanied by increased number of effector CD4^+^ and CD49b^+^ cells and decreased number of suppressor Tregs (Figure 2B). Moreover, MDSCs infiltrating MC38/shTGFβ1-1 tumors revealed higher expression of CD86 than MDSCs from control tumors. Since increased expression of CD86 costimulatory molecule on surface of MDSCs may indicate higher maturation stage and decreased suppressor activity of the cells, in the further experiments the immunosuppressive activity of MDSCs was estimated. CFSE-based proliferation assay showed that CD11b^+^ cells isolated from spleens of mice with s.c. growing MC38/shTGFβ1-1 tumors have not inhibited T cell proliferation. In the case, the mean fluorescence intensity of CFSE-labeled CD8^+^ and CD4^+^ cells was comparable to the values obtained for ConA-treated positive control (Figures 2C,D), whereas the CD11b^+^ cells isolated from control mice with MC38 or MC38/shN tumors showed high suppressor activity and significantly inhibited proliferation of spleen cells. Additionally, myeloid cells isolated from mice with MC38/shTGFβ1-1 tumors stimulated lymphocytes to produce proinflammatory cytokines, such as IFN-γ and IL-17, while CD11b^+^ cells from control groups induced secretion of higher amounts of suppressor IL-10. Due to low number of engrafted MC38/shTGFβ1-1 and MC38/shN tumors, no statistically significant differences were measured. However, these observations suggest that inhibition of TGF-β1 production in tumor microenvironment may have a major impact on abrogation of MDSC-dependent suppression.

### Intratumorally Injected LVs Transduced Primarily Myeloid Cells Infiltrating MC38 Tumors and Reduced Their Suppressive Activity

**Figure 3:**
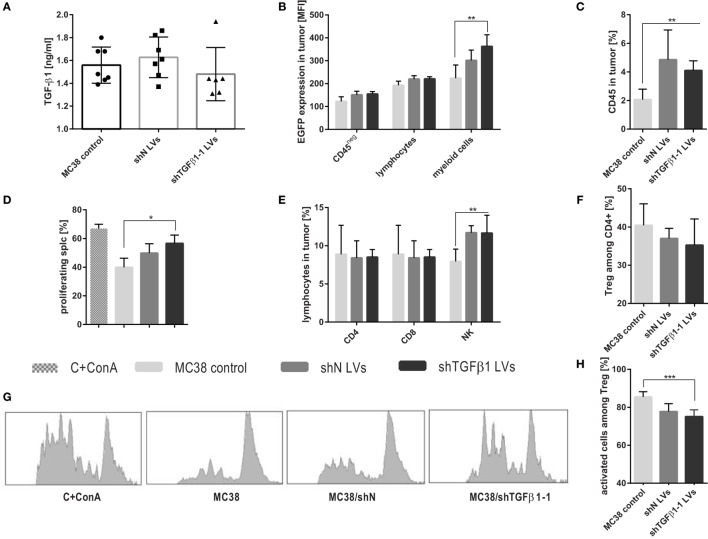
Intratumoral activity of shTGFβ1-1 lentivectors (LVs). Tumors were dissected and analyzed on the 6th day after the start of triple intratumoral injection of LVs. **(A)** TGFβ1 production by cells isolated from tumors. **(B)** EGFP expression in both in tumor cells and leukocytes and myeloid cells infiltrating into tumor. **(D,G)** Suppressor activity of tumor-derived myeloid cells estimated using CFSE-based proliferation assay. The mean fluorescence intensity (MFI) of whole population of splenocytes as well as proliferating CD8^+^ and CD4^+^ cells were presented as bar graph **(D)** and representative histograms **(G)**. As a positive control CFSE-labeled splc cultured with ConA were used. **(C,E,F,H)** The percentage of leukocytes and selected subpopulations of lymphocytes infiltrating into growing tumors collected on the 6th day after intratumoral injection of MC38/shTGFβ1-1 LVs cell line and adequate negative controls. To calculate the mean ± SD, at least six mice per group were analyzed. The differences between groups were estimated using non-parametric Kruskal–Wallis test followed by Dunn’s multi comparison test (*p < 0.05, **p < 0.01, ***p < 0.001).

Analysis of tumors, performed on the 6th day after the start point of the treatment, consisted of triple intratumoral injection of shN or shTGFβ1-1 LVs. It was shown that cells isolated from tumor tissue produced slightly lower amounts of TGF-β1 after application of shTGFβ1-1 LVs than cells isolated from shN LVs-treated mice or untreated control (Figure 3A). Nonetheless, the effect was not observed on the level of mRNA (data not presented). In order to check which cells in the tumor were preferentially transduced by LVs, we performed detailed analysis of immune cells infiltrating tumors. Since LVs encoded additionally EGFP, the changes in EGFP expression in particular cell subpopulations were measured. The obtained data showed only slight changes in EGFP expression in tumor cells and lymphocytes. The best effect of transduction was observed in myeloid cells. The statistically significant increase of EGFP expression in the cells was noted after treatment with shTGFβ1-1 LVs (Figure 3B). Among myeloid cell population, we identified TAM, DC, M-MDSC, and PMN-MDSC, which also showed increased expression of EGFP (data not presented). Moreover, myeloid cells isolated from tumors of mice treated with shTGFβ1-1 revealed decreased suppressor activity toward splenocytes obtained from healthy mice (Figures 3D,G; Figure S1 in Supplementary Material). The changes in myeloid cell activity together with reduced amount of TGF-β1 in tumor milieu presumably caused the reduction of Treg cell number and their activity (Figures 3F,H). In spite of increased number of leukocytes inside treated tumors, on the 6th day, we did not observed CTL influx, which indicates that antiviral response has not yet been induced (Figures 3C,E). On the other hand, after LVs treatment, we noted higher percentage of NK cells inside tumors than in control group. Obtained data show that myeloid cells are main target of intratumorally injected LVs. Moreover, it demonstrates that the total elimination of TGF-β1 from tumor microenvironment is not necessary to redirect immune response.

### Therapy with LVs and DCs Induced TGI and Increased Influx of Effector Cell into the Tumor

**Figure 4:**
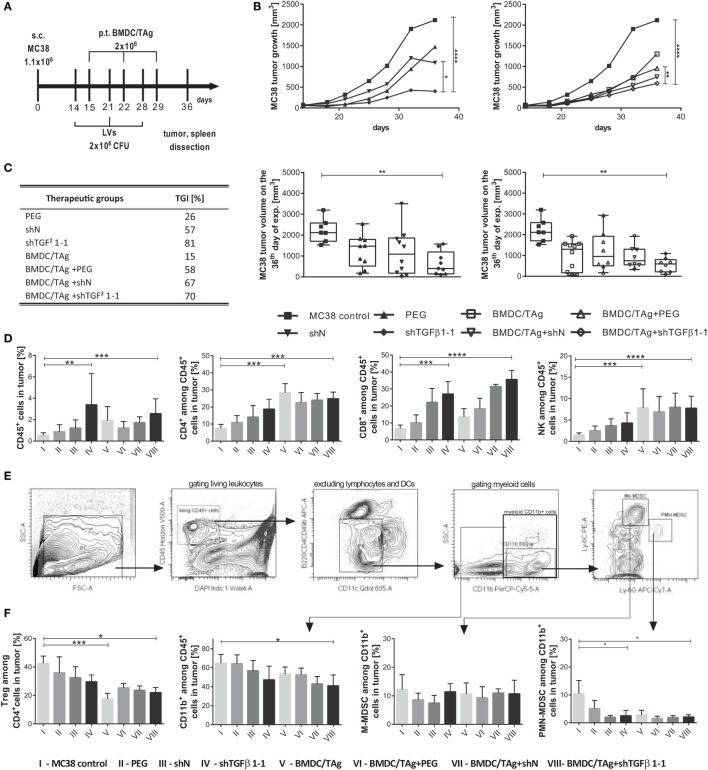
MC38 tumor growth and immune cell influx into tumor nodules after treatment with shTGF-β1-1 lentivectors (LVs) and BMDC/TAg. **(A)** The treatment scheme. Mice bearing advanced s.c. growing MC38 tumors were treated intratumorally (i.t.) with LVs carrying shTGF-β1-1 or scrambled short hairpin RNA sequence as a negative control (10^6^ CFU/mouse). The injections were repeated three times at weekly intervals. BMDC/TAg-based vaccine was administered s.c. 1 day after each LV injection. **(B)** Median of MC38 tumor growth after treatment with LVs or their combination with dendritic cells compared to untreated mice. As the control for LV preparations, the PBS containing aggregates of PEG with cell debri or microvesicles remaining in culture supernatant from untransduced HEK293T was applied. The differences between groups were estimated using Friedman test. The box graphs present the median of the tumor volume, calculated on the 36th day after MC38 tumor inoculation. **(C)** Tumor growth inhibition (TGI) calculated on the 36th day of experiment in relation to the group of untreated mice. **(D,E,F)** Flow cytometric analysis of immune cell influx in tumor nodules dissected on the 36th day of the experiment. **(D)** The percentage of effector CD4^+^, CD8^+^, and CD49b^+^ (NK) cells among leukocytes (CD45^+^) infiltrating into tumor nodules. **(E)** Representative dot plots demonstrating the method of identification of myeloid-derived suppressor cell (MDSC) subpopulations in tumors. **(F)** The percentage of suppressor Treg and M-MDSC or PMN-MDSC among myeloid CD11b^+^ cells infiltrating tumor. To calculate the mean ± SD, at least five mice per group were analyzed in one of the two independent experiments. The differences between groups were estimated using non-parametric Kruskal–Wallis test followed by Dunn’s multi comparison test (*p < 0.05, **p < 0.01, ***p < 0.001, ****p < 0.0001).

Mice with s.c. growing, advanced MC38 tumors (approximately 50 mm^3^ at the time of the therapy starting), were treated with lentiviral vectors carrying sequences of shRNA targeting TGF-β1 (shTGFβ1-1 LVs) or their combination with BMDC-based vaccines according to the scheme presented in the Figure 4A. Before application, the *ex vivo* differentiated BMDCs were stimulated with MC38 tumor lysate (BMDC/TAg). Control groups received lentiviral vectors carrying scrambled sequences of shRNA (shN LVs) or control of lentiviral preparations named here as PEG. The kinetics of MC38 tumor growth and the percentage of TGI calculated on the 36th day of experiment in relation to untreated MC38-bearing mice were presented in the Figures 4B,C. The obtained data showed that triple injection of lentiviral vectors silencing TGF-β1 significantly reduced MC38 tumor growth. In this case, TGI amounted to 81% and was considerably higher than in control PEG or shN groups reaching 26 and 56%, respectively. However, when shTGFβ1-1 LVs were applied in combination with BMDC/TAg, the TGI was lower than in the group treated with shTGFβ1-1 LVs (70%) and comparable with that obtained for the BMDC/TAg + shN group (67%).

The analysis of tumors dissected on the 36th day of the experiment confirmed increased influx of leukocytes (CD45^+^) into tumors after therapy. The highest number of the cells was observed in tumors of mice which received shTGFβ1-1 LVs and reached 3.4% in the shTGFβ1-1 group and 2.6% in the BMDC/TAg + shTGFβ1-1 group. The obtained values were more than fivefold higher in comparison to those noted for untreated MC38 control, and the differences were statistically significant (Figure 4D). It was observed that CD11b^+^ myeloid cells were the most numerous cell population of all the tumor-infiltrating leukocytes in the MC38 control group and amounted to 65%, whereas T lymphocytes and NK cells together constituted approximately 16% (the percentage of CD4^+^ cells amounted to 7.5%; CD8^+^—6.7% and CD49b^+^—1.5%; Figures 4D,F). These proportions were drastically changed after therapy. Generally, it was observed that treatment with LVs (either with shN or shTGFβ1-1) resulted in significantly increased number of CD8^+^ lymphocytes in the tumor nodules. The percentage of the cells in the group treated with shTGFβ1-1 LVs reached 27% and increased up to 36% after co-treatment with BMDC/TAg. However, the high CD8^+^ cell numbers were noted also in control groups treated with shN LVs and BMDC/TAg + shN LVs and amounted to 22 and 31%, respectively. This observation suggests that high infiltration of CD8^+^ lymphocytes was primarily connected with virus presence. The shTGFβ1-1 LVs treatment caused also enhanced influx of CD4^+^ and NK (CD49b^+^CD8^−^CD4^−^) cells into tumor nodules, which was additionally enlarged by BMDC/TAg treatment. However, in the group of mice injected only with BMDC/TAg, the numbers of CD4^+^ and NK cells were equally high as in the group treated with BMDC/TAg + shTGFβ1-1 LVs. The data indicate that the enhanced infiltration of Th and NK cells was induced presumably by BMDC/TAg and not by LVs carrying shTGFβ1. Apart from effector cells, suppressor Tregs and MDSCs were also detected in the tumor tissue. In the control group, Tregs (CD4^+^CD25^+^FoxP3^+^) constituted 43% of all the CD4^+^ cells. Their number significantly declined after therapy and amounted to 29% for shTGFβ1-1 LVs and 22% for BMDC/TAg + shTGFβ1-1 LVs-treated mice. Interestingly, the lowest Treg number (17%) was observed in the group which received only BMDC/TAg (Figure 4F). After therapy, we also observed decrease in myeloid CD11b^+^ cell number. Together with the decrease of CD11b^+^ population in tumors, the proportion of MDSCs subpopulations underwent changes. The identification of particular subpopulations of MDSCs was performed according to the procedure outlined in Figure 4E. Generally, after treatment with LVs (either with shN or shTGFβ1-1), the numbers of PMN-MDSC constituted approximately 2% of all CD11b^+^ cells in tumor and were significantly lower than in the MC38 control (10%; Figure 4F). However, no differences between shTGFβ1-1 and BMDC/TAg + shTGFβ1-1 groups were noted.

**Figure 5:**
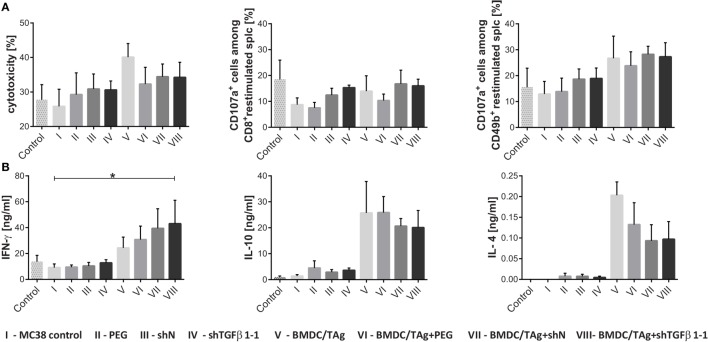
Activity of spleen cells isolated from MC38-bearing mice treated with shTGF-β1 lentivectors and bone marrow-derived dendritic cell (BMDC)/TAg. Splenocytes obtained from treated mice on the 36th day of experiments were restimulated *in vitro* in the presence of MC38 cells for 5 days. **(A)** Cytotoxic activity of restimulated cells toward MC38 tumor (the specific killing of DiOC_18_-labeled MC38 tumor cells was assessed by PI incorporation flow cytometry measurement) and the percentage of CD107a positive cells among cytotoxic CD8^+^ and CD49b^+^ cells; **(B)** ability of restimulated splenocytes to produce IFN-γ, IL-10, and IL-4. Cytokine concentrations in supernatants collected after 5 days of restimulation were measured by ELISA. As a control, the splenocytes from healthy mice were used. To calculate the mean ± SD at least five mice per group were analyzed in one of the two independent experiments. The differences between groups were estimated using non-parametric Kruskal–Wallis test followed by Dunn’s multi comparison test (*p < 0.05).

The next step of our investigation was to determine the effect of the therapy on the induction of systemic antitumor immune response. The highest cytotoxic activity (40%, 1.6-fold higher in comparison to MC38 control; Figure 5A) was noted in the group of mice receiving BMDC/TAg. Moreover, obtained data demonstrate that BMDC/TAg application induced cytotoxicity depended on NK cells. All groups receiving BMDC-based vaccine were characterized by the highest percentages of CD49b^+^CD107a^+^ cells, which were approximately 1.5-fold higher than in the MC38 control (Figure 5A; Figure S2 in Supplementary Material). While, LVs encoding shTGFβ1-1 were significantly less effective in activation of cytotoxic cells and in the group treated only with shTGFβ1-1 LVs the cytotoxicity was 30%. TGF-β1 silencing also did not enhance BMDC/TAg ability to activate cytotoxic cells. The LV-treated groups, both shN and shTGFβ1-1 LVs, showed increased number of CD8^+^CD107a^+^ cells when compared to MC38 control (Figure 5A; Figure S2 in Supplementary Material). During further tests, the ability of splenocytes from particular groups to produce cytokines was also examined. The increase of IFN-γ production was observed only in groups treated with BMDC/TAg, whereas the highest concentration of IFN-γ in supernatants was noted in the group treated with BMDC/TAg + shTGFβ1-1 LVs. The value was more than fourfold higher than that obtained in MC38 control group and almost twofold higher than these observed in the BMDC/TAg-treated group (Figure 5B). Apart from IFN-γ, the IL-10 and IL-4 were also detected in collected supernatants. The treatment with BMDC/TAg considerably increased the ability of spleen cells to produce Th2-type cytokines. The combination of BMDC/TAg with LVs (both shN and shTGFβ1-1) resulted in decreased production of IL-10 and IL-4 inducing stronger Th1 signal. However, the effect was caused more possibly by the lentivirus presence than by TGF-β1 reduction. Nevertheless, taking into consideration all tested cytokines, the obtained data suggests that BMDC-based vaccines are significantly more potent in induction of Th1-type response when TGF-β1 concentration in tumor microenvironment is reduced.

### Pretreatment with Low Dose of CY followed by Application of shTGFβ1 LVs and BMDC/TAg Significantly Enhanced the Antitumor Effect of the Genoimmunotherapy

**Figure 6:**
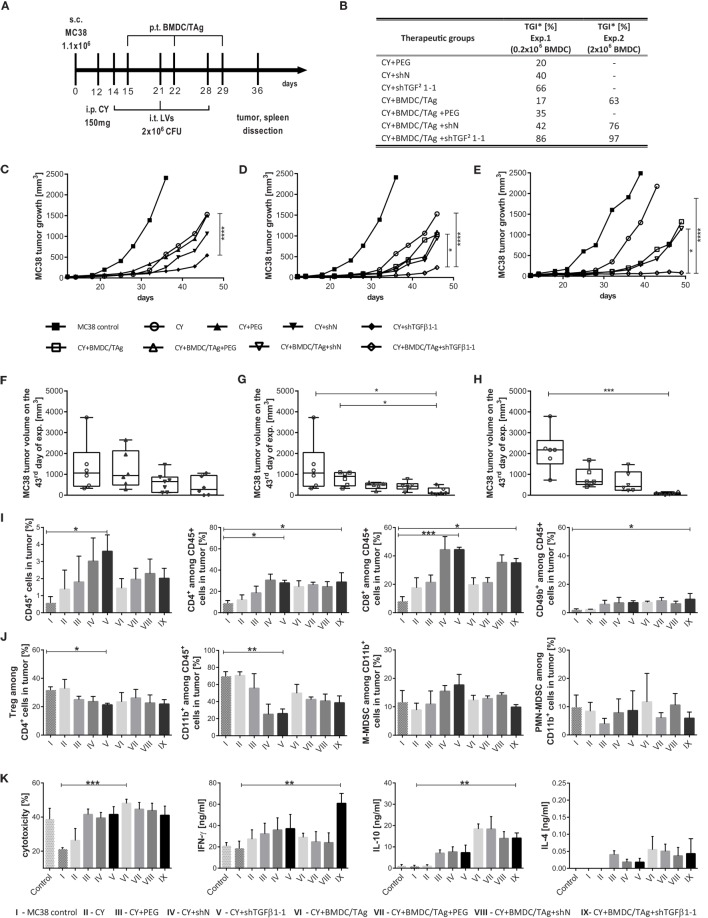
MC38 tumor growth and immune cell activity after combined therapy with low dose of clophosphamide (CY) and multiple injections of shTGF-β1 lentivectors (LVs) followed by BMDC/TAg vaccination. **(A)** The treatment scheme. Mice bearing advanced s.c. growing MC38 tumors were treated with single dose of CY (150 mg/mouse) and then injected i.t. with LVs carrying shTGF-β1-1 or scrambled short hairpin RNA sequence as a negative control (10^6^ CFU/mouse). The LV injections were repeated three times at weekly intervals. BMDC/TAg-based vaccine was administered s.c. 1 day after each LV injection. **(B)** Tumor growth inhibition (TGI) calculated on the 43rd day of experiment in relation to the CY-treated group of mice. **(C,D)** Kinetics of MC38 tumor growth after treatment with CY and LVs or their combination with 0.2 × 10^6^ bone marrow-derived dendritic cell (BMDC)/TAg/mouse; **(E)** Kinetics of MC38 tumor growth after treatment with CY, LVs, and BMDC/TAg (2 × 10^6^ cells/mouse). The differences between groups were estimated using Friedman test. The box graphs present the median of the tumor volume, calculated on the 43rd day after MC38 tumor inoculation after treatment with CY and LVs **(F)** or their combination with 0.2 × 10^6^ BMDC/TAg/mouse **(G)** or with 2 × 10^6^ BMDC/TAg/mouse **(H)**. **(I,J)** Flow cytometric analysis of immune cells in tumor nodules dissected on the 36th day of the experiment. **(I)** The percentage of effector CD4^+^, CD8^+^, and CD49b^+^ (NK) cells among leukocytes (CD45^+^) infiltrating into tumor nodules. **(J)** The percentage of suppressor Treg and M-MDSC or PMN-MDSC among myeloid CD11b^+^ cells infiltrating tumor. **(K)** Cytotoxic activity of splenocytes and their ability to produce IFN-γ, IL-10, and IL-4. Splenocytes isolated from mice on the 36th day of experiment were restimulated for 5 days in the presence of MC38 cells. The concentration of cytokines was measured in supernatant collected after restimulation using ELISA. The specific killing of DiOC_18_-labeled MC38 tumor cells by restimulated splenocytes was assessed by PI incorporation flow cytometry measurement. As a control, the splenocytes from healthy mice were used. To calculate the mean ± SD, at least five mice per group were analyzed in one of the two independent experiments. The differences between groups were estimated using non-parametric Kruskal–Wallis test followed by Dunn’s multi comparison test (*p < 0.05, **p < 0.01, ***p < 0.001, ****p < 0.0001).

In the therapeutic scheme, low dose of CY (CY; 150 mg/kg) was applied 2 days before treatment with shTGFβ1-1 LVs and BMDC/TAg (Figure 6A). Two kinds of experiments with different number of BMDC/TAg [0.2 × 10^6^ cells/injection (Figures 6C,D,F,G) or 2.0 × 10^6^ cells/injection (Figures 6E,H)] were performed. The kinetics of MC38 tumor growth as well as TGI calculated on the 43rd day of experiment in relation to CY-treated group were presented in Figures 6B–H. It was observed that CY followed by triple injection of shTGFβ1-1 LVs significantly reduced MC38 tumor growth. In this case, TGI amounted to 66% and was significantly higher than in controls treated with PEG (20%) and shN (40%). Treatment with CY and BMDC/TAg + shTGFβ1-1 LVs induced the highest TGI in the experiment and the TGI rate amounted to 86%. The kinetic of the MC38 tumor growth showed statistically significant difference between the group and CY and PEG control group (Figures 6D,G). The increase of the BMDC/TAg number up to 2 × 10^6^ cells/inj. contributed to additional increase in the efficacy of the therapy. In the second experiment, the TGI in the CY + BMDC/TAg + shTGFβ1-1 group reached 97% and was significantly higher than in the BMDC/TAg (63%) and BMDC/TAg + shN LVs (76%) treated groups (Figures 6B,E,F).

Further analysis of tumors showed that CY application followed by shTGFβ1-1 LVs injections induced the highest influx of leukocytes into tumor nodules. In this case, the percentage of CD45^+^ cells reached 3.6% and was sixfold higher than in the MC38 control. Interestingly, the number of leukocytes in the groups of mice receiving BMDC/TAg was significantly lower and ranged from 1.4% for CY + BMDC/TAg to 2.3% for CY + BMDC/TAg + shTGFβ1-1 group (Figure 6I). Also in this treatment scheme, LV injection resulted in very high influx of CD8^+^ cells into tumors. The highest number of CD8^+^ cells among leukocytes (approximately 44%) was observed in tumors of CY + shN and CY + shTGFβ-1 groups. Lower values for the cell population (approximately 35%) were noted in CY + BMDC/TAg + shN and CY + BMDC/TAg + shTGFβ-1 groups. In the remaining control groups, the percentage of CD8^+^ cells did not exceed 20%. Moreover, LVs as well as dendritic cells induced higher infiltration of CD4^+^ and NK cells when compared to the untreated MC38 or CY-treated controls. However, no differences between these therapeutic groups were observed and the approximate percentages of CD4^+^ and NK cells amounted to 25 and 7,5%, respectively (Figure 6I). The influence of applied therapy on the number of suppressor Tregs and MDSCs in tumors was also examined. On the 24th day after treatment with CY, the effect of CY on reduction of Treg number in tumor tissue has been no longer noted. However, the percentages of Tregs after treatment with CY + shTGFβ1-1 LVs (21%) and CY + BMDC/TAg + shTGFβ1-1 (21%) were significantly lower than in the MC38 control and CY-treated mice (32%; Figure 6J). Additionally, after application of shN LVs the numbers of Tregs were also reduced and only slight differences between groups receiving shTGFβ1-1 and shN were noted. The CY, LVs, and BMDC/TAg combined therapy resulted in considerable reduction of CD11b^+^ cell influx into tumor nodules in comparison to MC38 control. But in this case as well, only small differences in the percentage of CD11b^+^ between shTGFβ1-1 LVs- and shN LVs-treated group were detected. The data suggest that decrease of Treg and CD11b^+^ numbers in tumor nodules was induced by LVs and did not depend on TGF-β1 elimination. In contrast to therapy without CY, in this treatment scheme, the changes in the number of M-MDSCs in particular groups were observed. Interestingly, the application of CY + shTGFβ1-1 LVs caused an increase of M-MDSC number in comparison to MC38 control. In the group, M-MDSCs constituted 18% of CD11b^+^ cell population and was 1.5-fold higher than in the MC38 control. On the other hand, treatment with CY + BMDC/TAg + shTGFβ1-1 LVs resulted in a slight decrease of M-MDSC number as compared to the MC38 control (Figure 6J). The similar tendency was observed in the case of PMN-MDSCs. Their number after treatment with CY + BMDC/TAg + shTGFβ1-1 LVs was lower than in the MC38 control as well as CY + shTGFβ1-1 LV-treated mice (Figure 6J).

The cytotoxic activity and cytokine production by restimulated splenocytes from mice treated with CY, LVs, and BMDC/TAg were measured in order to determine a systemic antitumor immune response. As in previous experiments, the highest cytotoxic activity of splc toward MC38 cells were observed after treatment with CY + BMDC/TAg and amounted to 48% (Figure 6K). Lower values, approximately 41%, were obtained for groups which received shTGFβ1-1 LVs and were comparable to those receiving shN LVs controls. Although, the obtained values were more than twofold higher compared to the MC38 control group, there was no visible effect of the TGF-β1 elimination on the systemic induction of specific CTLs. However, the influence of the CY + BMDC/TAg + shTGFβ1-1 LVs treatment on the IFN-γ production was noted. In the group, production of IFN-γ was considerably higher than in remaining groups. The LV treatment (both shTGFβ1-1 and shN) caused changes in the IL-10 and IL-4 production. As in the case of the therapy without CY, in this scheme of treatment, the concentrations of IL-10 and IL-4 in supernatants were lower after application of CY + BMDC/TAg + shTGFβ1-1 LVs or CY + BMDC/TAg + shN LVs than in the BMDC/TAg group (Figure 6K). The obtained data confirmed previous observations demonstrating an activation of Th1-type response after combined therapy with BMDC/TAg-and shTGFβ1-1 LVs.

## Discussion

In the following work, the enhancement of the antitumor activity of dendritic cell-based vaccines by previous elimination of TGF-β1 from murine colon carcinoma microenvironment was evaluated. The expression of TGF-β1 was silenced using RNAi technique, which gained a great popularity in the recent years because of its high specificity and wide range of applicability. There are some reports showing the antitumor potential of siRNA molecules. However, due to their low stability in the tumor site frequent inoculations were necessary to obtain the desired effect (17). In the article, we described, for the first time, the effectiveness of intratumoral application of lentiviral vectors carrying shRNA sequences specific for TGF-β1.

In the first step of our study, we carried out experiments allowing us to choose the most effective shRNA sequence targeting TGF-β1. The MC38 cells transduced with shTGFβ1-1 were characterized by the lowest production of TGF-β1 and increased immunogenicity. The obtained result is in line with previous studies by Wei and coworkers (29). As in our study, they used lentiviral vectors to introduce shTGF-β1 sequences into tumor cells and reported that dendritic cells stimulated with homogenates from human ovarian carcinoma cells with silenced expression of TGF-β1 were more efficient in activation of antigen-specific CTLs. Moreover, TGF-β1 silencing promoted expression of known ovarian carcinoma antigens as mesothelin and HE4. Thus, increased immunogenicity of transduced tumor cells could be triggered by reduced amount of TGF-β1 in homogenate as well as by increased expression of tumor antigens on transduced cells. In our study, all MC38/shTGFβ1 cell lines induced increase of costimulatory molecule expression on the surface of BMDCs which is associated with maturation of the cells. The obtained data indicate that inhibition of TGF-β1 production significantly reduced the immunosuppressive effect of MC38 cells on dendritic cells *in vitro*, at the same time increasing their ability to induce specific Th1/Tc1 type immune response. After subcutaneous inoculation, the MC38/shTGFβ1-1 cells were characterized by significantly lower tumorigenicity than wild MC38 cells. The *ex vivo* analysis of formed MC38/shTGFβ1-1 tumor nodules revealed higher infiltration of leukocytes accompanied by increased numbers of Th, CTL, and NK effector cells and diminished numbers of Treg and PMN-MDSC immune suppressors. Due to increased expression of CD86 on surface of the MC38/shTGFβ1-1 tumor-infiltrating PMN-MDSC and M-MDSC, the suppressor activity of splenic myeloid CD11b^+^ cells was also evaluated. The obtained data showed that tumor cells with inhibited production of TGF-β1 were not able to induce the MDSC-dependent suppression (Figure 2). Similar observations were described by Llopiz and coworkers, which noted depleted number of splenic MDSC after treatment with TGF-β peptide inhibitors (30). Although, the chemoattractant functions of TGF-β1 facilitating the recruitment of macrophages to the site of inflammation and its role in polarization of M1-type macrophages toward M2 are well known, the direct influence of TGF-β1 on MDSC activity still has not been well described (31). Liu and coworkers reported that TGF-β1 can regulate MDSC function by altering miR494 expression (32). Researchers noted also that suppressor activity of MDSC may depend on TGF-β1 presence in tumor-derived exosomes (33). It seems probable that the mechanism with TGF-β1-delivering exosomes is responsible for inhibition of suppressor activity of splenic MDSC in our model. Indeed, MC38/shTGFβ1-derived exosomes cannot deliver TGF-β1 to other cells since the production of the cytokine in those cells is inhibited. However, it needs further investigations.

The main purpose of the work was to apply the LVs encoding shRNA sequences specific for TGF-β1 as an adjuvant enhancing the effectiveness of DC-based immunotherapy. The LVs were inoculated intratumorally, in the total number of 2 × 10^6^ particles per injection, 1 day before each injection of BMDC/TAg-based vaccine. It was documented that after cutaneous injection, free viruses are rapidly eliminated. However, transduced cells with LV-derived transgene may accumulate and proliferate in the site of injection even for more than 3 weeks after immunization (34–36). After intratumoral inoculation, LVs are able to transduce both tumor cells and other cells infiltrating tumor including immune cells. Upon transduction, tumor cells gain not only transgene but also viral antigens and in the consequence, they are better recognized by immune cells. Tumor-infiltrating DCs may not only directly present viral antigens in the context of the MHC class I molecules to CTLs and induce antiviral response but also cross-present exogenous antigens from dying tumor cells and activate antitumor response (37, 38). Finally, all transduced cells can be recognized and killed by immune cells. However, even 3-week lasting downregulation of TGF-β1 in the injection site is crucial for proper functioning of inoculated peritumorally BMDC/TAg-based vaccines. Due to limited tumor penetration by LV particles, in our therapeutic scheme, we applied three separate intratumoral injections of LVs, thereby increasing the number of transduced cells inside the tumor. In the first of presented here therapeutic experiment the influence of treatments with LVs targeting TGF-β1, applied alone or in combination with dendritic cells, on the MC38 TGI was similar (Figure 4). It seems probable that in this scheme of treatment LVs targeting TGF-β1 were the most important component of the therapy. In the second proposed scheme, we applied the pretreatment with low dose of CY (Figure 6). In this case, the effect of the therapy consisted of CY + BMDC/TAg + shTGFβ1-1 LVs was significantly better than application of CY + BMDC/TAg or CY + shTGFβ1-1 LVs. Moreover, the difference between CY + BMDC/TAg + shN LVs or CY + BMDC/TAg + shTGFβ1-1 LVs groups was statistically significant. It should be also noted that the final effect of the therapy depended on the number of dendritic cells in applied cell-based vaccines, namely, increasing the number of BMDC/TAg in vaccine from 0.2 to 2.0 × 10^6^ cells/injection considerably prolonged the MC38 tumor growth delay. Comparing those two schemes of treatment, it seems probable that pretreatment with CY was crucial for dendritic cell-based vaccine to adapt in the new environment. The immunoregulatory role of low doses of CY is well described in scientific literature. CY can act as stimulator of effector immune cells as well as it can decrease the number of Treg, thereby reducing the amount of TGF-β1 in tumor microenvironment. Moreover, CY induce lymphodepletion creating in consequence an “empty space,” which can be finally colonized by peritumorally inoculated dendritic cells with increased ability to regenerate and actuate a specific immune response (27, 39). However, the pretreatment with CY, without TGF-β1 elimination, was not enough for proper functioning of inoculated dendritic cells. The application of CY + BMDC/TAg caused minor therapeutic effect compared to group of mice receiving CY + BMDC/TAg + shTGF1-1 LVs. There is a possibility to apply metronomic treatment with CY in order to sustain the positive effect of CY on antitumor potential of BMDC/TAg. Nevertheless, the elimination of TGF-β1 using specific LVs seems to be a better solution since multiple application of CY causes increased accumulation of MDSC in tumor (40). It should be also stressed that in the scheme of treatment, the intratumoral application of shTGFβ1 LVs induced significantly higher TGI than that obtained the scheme with an application of antibodies against TGFβ1 (unpublished observations).

The *ex vivo* analysis of local and systemic antitumor response revealed that the mechanism of action of the presented therapeutic schemes was similar. Our data showed that application of LVs (shTGFβ1-1 as well as control shN) induced enhanced influx of leukocytes into tumor including high number of CTLs. Despite this, restimulated splenocytes obtained from LV-treated mice have not revealed significant differences in cytotoxic activity toward MC38 cells. The observations indicate that an increased influx of CTLs into tumor nodules may be associated with induction of antiviral response and are in line with work of Hotblac’s team demonstrating that the third-generation lentiviral system can deliver LV-derived molecule responsible for DC activation followed by induction of specific antiviral CTL response (38). Apart from the CTL activation, we observed also influence of applied therapy on IFN-γ production by restimulated splenocytes. Increased level of the cytokine was generally connected with application of BMDC/TAg. However, the highest concentration of IFN-γ was noted in groups which received both BMDC/TAg and shTGFβ1-1 LVs. It means that elimination of TGF-β-1 from tumor milieu supports dendritic cells in activation of systemic Th1-type immune response. Moreover, we observed influence of these treatments on changes of the suppressor cell number in tumor tissue. As expected, after therapy with shTGF-β1 LVs the lowest number of Treg was observed. However, in the case of MDSC, especially M-MDSC, the effect was dependent on the pretreatment with CY, namely, in the scheme of treatment without CY, the influence of applied therapy on the number of M-MDSC in tumor nodules was not observed. On the other hand, after singular dosage of CY followed by BMDC/TAg + shTGFβ1-1 LVs treatment, the number of the cells in tumor nodules was lower than in others groups. It seems probable that shTGFβ1-1 LVs application supports the CY-induced elimination of MDSCs from tumor microenvironment.

Concluding, the application of shTGFβ1 LVs alone or in combination with DC-based vaccines is not sufficient for long-lasting elimination of suppression in tumor. However, simultaneous reduction of TGF-β1 in tumor microenvironment and its remodeling by pretreatment with a low dose of CY facilitates the settlement of peritumorally inoculated DCs and supports them in restoration and activation of a potent antitumor response. Moreover, the obtained data provide a new insight into the way of acting of shTGFβ1-1 LVs that were delivered directly to the tumor microenvironment. The LVs seem to play a dual role in the proposed scheme of treatment. First, their application diminished the suppression in tumor and simultaneously it ensured a better recognition of tumor cells by immune cells inducing coexisting antiviral response.

## Ethics Statement

This study was carried out in accordance with the recommendations of The Act on the protection of animals used for scientific or educational purposes (Art. 30 Act 1 Item 1 of January 21, 2005) issued by Ministry of Science and Information. The protocol was approved by the First Local Ethics Committee for Experiments with the Use of Laboratory Animals, Wroclaw, Poland.

## Author Contributions

JR: substantial contribution to the conception and the design of the research, planning and performing experiments, data interpretation, and drafting the manuscript. NA: planning and performing *in vitro, in vivo*, and *ex vivo* experiments and data analysis. AS and JM: planning and performing *ex vivo* experiments. EP-P: planning and performing *in vivo* experiments. All authors reviewed the manuscript and approved its final version.

## Conflict of Interest Statement

The authors declare that the research was conducted in the absence of any commercial or financial relationships that could be construed as a potential conflict of interest.

## Supplementary Material

Figure S1 Intratumoral activity of shTGFβ1-1 lentivectors. Suppressor activity of tumor-derived myeloid cells estimated using CFSE-based proliferation assay. The percentage of proliferating splenocytes was presented as bar graph. As a positive control CFSE-labeled splc cultured with ConA were used. To calculate the mean ± SD at least six mice per group were analyzed. The differences between groups were estimated using non-parametric Kruskal–Wallis test followed by Dunn’s multi comparison test (**p* < 0.05).

Figure S2 Activity of spleen cells isolated from MC38-bearing mice treated with shTGF-β1 lentivectors and BMDC/TAg. Splenocytes obtained from treated mice on the 36th day of experiments were restimulated *in vitro* in the presence of MC38 cells for 5 days. **(A)** Representative histograms showing expression of CD107a molecule on surface of cytotoxic CD8^+^ and CD49b^+^ cells.

## Abbreviations

BMDCs, bone marrow-derived dendritic cells; MC38, murine colon carcinoma; TAg, tumor antigen; CY, cyclophosphamide; MDSCs, myeloid-derived suppressor cells; Tregs, T regulatory lymphocytes; LVs, lentiviral vectors; shTGF-β1, shRNA sequences targeting TGF-β1 mRNA.
